# Dasatinib inhibits actin fiber reorganization and promotes endothelial cell permeability through RhoA‐ROCK pathway

**DOI:** 10.1002/cam4.1019

**Published:** 2017-03-18

**Authors:** Swapan K. Dasgupta, Anhquyen Le, K. Vinod Vijayan, Perumal Thiagarajan

**Affiliations:** ^1^ Center for Translational Research on Inflammatory Diseases (CTRID)Michael E. DeBakey Veterans Affairs Medical CenterDepartment of PathologyBaylor College of MedicineHoustonTexas; ^2^ Center for Translational Research on Inflammatory Diseases (CTRID)Michael E. DeBakey Veterans Affairs Medical CenterDepartment of MedicineBaylor College of MedicineHoustonTexas

**Keywords:** Actin fibers, dasatinib, endothelial permeability, tyrosine phosphorylation

## Abstract

Treatment with dasatinib, a tyrosine kinase inhibitor, is associated with edema, pleural effusion, and pulmonary edema. We investigated the effect of dasatinib on the barrier function of human microvascular endothelial cells‐1 (HMEC‐1) in vitro and in vivo. The permeability of HMEC‐1 to fluorescein isothiocyante (FITC)‐dextran increased in Transwell chambers within 5 min following the addition of therapeutic concentrations of dasatinib. The change in permeability was associated with increased activation of RhoA GTPase and its effector Rho‐associated coiled‐coil kinase 1(ROCK1). RhoA inhibitor C3 transferase almost completely inhibited dasatinib‐induced increase in permeability. Under similar conditions, imatinib had no effect on permeability or activation of RhoA. Since integrin‐induced cell spreading suppresses RhoA activation, we examined the effect of dasatinib on cell spreading on fibronectin substrate. Dasatinib impaired endothelial cell spreading in a concentration‐dependent manner and induced disorganization of actin fibers. Tyrosine kinases play an essential role in transmitting signals from integrins to RhoA and we examined tyrosine phosphorylation of several cytoskeletal proteins. Dasatinib markedly inhibited tyrosine phosphorylation of p130 Crk‐associated substrate (p130cas), paxillin and vinculin. These results suggest that the inhibition of tyrosine phosphorylation of the focal adhesion plaque components by dasatinib may alter the assembly of actin fibers resulting in the activation of RhoA/ROCK pathway. Consistent with these findings, dasatinib‐induced increase in the permeability was blocked by ROCK inhibitor y27632. In vivo administration of y27632, significantly inhibited the dasatinib‐induced extravasation of Evans blue in mice and dasatinib‐induced increase in microvascular permeability was attenuated in ROCK1‐deficient mice. These findings suggest that ROCK inhibitors could serve as therapeutic modalities to ameliorate the dasatinib‐induced pulmonary changes.

## Introduction

The balanced translocation of 5′ segment of the breakpoint cluster region (BCR) gene on chromosome 22 to the 3′ segment of the Abelson leukemia virus (ABL) gene on chromosome 9 in chronic myeloid leukemia (CML), leads to the formation of the BCR‐ABL hybrid fusion gene, which has constitutively upregulated tyrosine kinase activity. Imatinib mesylate, a small molecule developed specifically to inhibit BCR‐ABL kinase activity, has revolutionized the treatment of CML 1. Despite imatinib's overwhelming success, 20% of patients develop resistance to imatinib due to point mutations in the BCR–ABL kinase domain 2. Dasatinib is a second generation of tyrosine kinase inhibitors (TKI) that was developed to overcome the imatinib resistance 3. However, in addition to BCR‐ABL kinase, dasatinib inhibits other tyrosine kinases such as Src family kinases (SFKs), stem cell factor receptor (c‐KIT), and platelet‐derived growth factor receptor (PDGFR) 4 leading to its unique side effects. Clinically, the most common nonhematologic side effects seen in 20% of patients treated with dasatinib are peripheral edema, pleural effusion, and pulmonary hypertension 5, 6.

Increased permeability of microvasculature is the basis of peripheral edema, pleural effusion, and lung edema seen in many conditions, including sepsis and acute respiratory distress syndrome 7. The actin cytoskeletal network plays a major role in the maintenance of the endothelial barrier function, as it regulates the integrity of the endothelial lining 8. Here, we investigated the effect of dasatinib at therapeutic concentrations 9 on endothelial cells.

## Materials and Methods

### Reagents

Anti‐p130cas, anti‐myosin light chain kinase (MLC), anti‐phospho p130Cas (Y165), and anti‐phosphopaxillin (Y118) antibodies were from Cell Signaling Tech., Danvers, MA, USA. Anti‐paxillin antibody was from ECM Biosciences LLC, Versailles, KY, USA, Anti‐FAK (focal adhesion kinase), anti‐vinculin, anti‐phosphotyrosine, and anti‐phospho MLC (Thr18/Ser19 serine) antibodies were from Santa Cruz Biotechnology, Dallas, TX, USA. Anti‐phospho‐vinculin (Y1065) was from abcam, Cambridge, MA, USA. Alexa Fluor 546‐conjugated secondary antibody, Alexa Fluor 488‐conjugated phalloıdin, Fluorescein isothiocyante (FITC)‐labeled dextran and ROCK inhibitor y27632 were purchased from Thermo Fisher Scientific, Waltham, MA, USA. RhoA inhibitor I, (purified C3 Transferase covalently linked to a proprietary cell penetrating moiety), was purchased from Cytoskeleton Inc., Denver, CO, USA. HMEC‐1 (human microvascular endothelial cell‐1) 10 was obtained from the Centers for Disease Control and Prevention (Atlanta, GA) and cultured in vascular cell basal medium supplemented with endothelial cell growth factor kit (Invitrogen, Waltham, MA, USA). Dasatinib was obtained from Selleck Chem., Houston, TX, USA.

### Mice

The generation and maintenance of homozygous ROCK1‐deficient (ROCK1^−/−^) mice in an FvB background has been described previously 11. ROCK1^−/−^ mice are viable and morphologically indistinguishable from their wild‐type littermates. However, the number of ROCK1^−/−^ offspring from heterozygous parent mice was significantly below the normal Mendelian distribution. All animals were treated in accordance with the protocol approved by the Animal Care and Use Committee (IACUC) of Baylor College of Medicine.

### Measurement of microvascular permeability in vitro

Endothelial permeability assays were performed with endothelial cells grown on Transwell polycarbonate membrane inserts (Costar Inc., 6.5 mm in diameter, 3 *μ*m pore size). A cell suspension (2 × 10^6^ cells) was added to the upper chambers of the Transwell apparatus. After cells were confluent (usually 24–48 h after seeding), the medium was carefully replaced by the serum‐free medium 199. FITC‐Dextran was added to the upper chamber (to a final concentration of 1 mg/mL) followed by dasatinib (0–100 ng/mL) or vehicle [dimethyl sulfoxide (DMSO)]. At various time intervals (0–120 min), the inserts were carefully lifted, and the medium in the lower chamber was stirred and transferred (each 75 *μ*L) to a 96‐well plate. A typical experiment was performed in quadruplicate. The fluorescence intensity of the samples was measured in a 96‐well plate fluorescence reader (Synergy MX, BioTek, Winooski, VT, USA) using excitation at 495 nm and emission at 520 nm wavelength. In some experiments, the monolayers were incubated with ROCK1 inhibitor y27632 for 10 min before the addition of dasatinib.

### RhoA‐ROCK activation assay

A RhoA activity assay was performed and quantified using the RhoA activation assay kit according to the manufacturer's instructions (Millipore, Massachusetts, USA). Briefly, HMEC‐1 were incubated with dasatinib (100 ng/mL) solubilized in Tris‐buffered saline containing 0.25% deoxycholic acid, 1% NP‐40, 1 mmol/L EDTA, and a phosphatase/protease inhibitor cocktail mixture (Thermo Fisher Scientific, Waltham, MA, USA). At various time intervals, cells were lysed and the cell lysate was incubated with Rhotekin‐linked agarose beads at 4°C for 60 min. The beads were washed with wash buffer and denatured. Active RhoA (GTP‐bound Rho) was detected by western blotting with an anti‐RhoA antibody.

### Spreading of endothelial cells

Confluent layers of endothelial cells were trypsinized, trypsin neutralized with fetal calf serum, washed and incubated with various concentrations of dasatinib (0–100 ng/mL) or vehicle control (DMSO 0.1%). Cells were seeded on fibronectin‐coated glass coverslips in 24‐well plates at a density of 10,000 cells per well in serum‐free basal medium. After 60 min at 37°C, the cells were fixed in methanol and stained with eosin. The images are taken with a microscope using 100× magnification. Average cell spreading or cell area was quantified using the Image J software 12. For the assessment of actin fibers, subconfluent endothelial cells on fibronectin‐coated coverslips were fixed with 4% paraformaldehyde in phosphate‐buffered saline (PBS) for 10 min at 20°C and washed thrice with PBS. Cells were permeabilized with 0.1% Triton‐X‐100 (Sigma‐Aldrich, St. Louis, MO, USA) in PBS for 5 min, washed with PBS, and incubated with anti‐FAK (for adhesion plaques) at 1:50 dilution overnight at 4°C. Following washing, cells were incubated with Alexa Fluor 546‐conjugated secondary antibody (to detect FAK) and Alexa Fluor 488‐conjugated phalloıdin (for actin) for 1 h at room temperature. Cells were washed, mounted on a glass slide and examined under a confocal microscope (Nikon Ecliopse TE 2000‐E) with 1000× magnification.

### Phosphorylation of cytoskeletal proteins

HMEC‐1 were incubated with dasatinib for various time intervals and solubilized in cell lysis buffer. Aliquots (40 *μ*g of protein) were separated on SDS‐PAGE in 8–16% gels, transferred to PVDF membranes, immunoblotted using phosphotyrosine‐specific antibodies to p130cas, paxillin, or vinculin and appropriate HRP‐conjugated secondary antibodies and analyzed in Odyssey Fc Dual‐Mode Imaging System (Li‐Cor Biosciences, Lincoln, Nebraska, USA). The blots were stripped and reprobed with antibodies to nonphosphorylated p130cas, paxillin, ABL, or vinculin. For phosphorylation of MLC, the blots were probed with anti‐phospho‐MLC (Thr18, Ser19) followed by total anti‐MLC antibodies. Band intensities were quantified using Image J software. The densitometry values of the phosphoblots were divided by the respective value of total protein to get the ratio of phosphoprotein to total protein. The ratio obtained with DMSO control was arbitrarily normalized to 1 and the data for dasatinib treatment were expressed as fold change. The phosphorylation status of FAK was assessed by immunoprecipitating FAK from cell lysate and immunoblotting the FAK immunoprecipitate with anti‐phosphotyrosine and anti‐FAK antibodies.

### In vivo lung permeability

FvB mice were given an intraperitoneal injection of y27632 (10 mg/kg) or saline (5 mice in each group). After 1 h, dasatinib 10 mg/kg was given intraperitoneally. After 4 h, Evans blue (2 mg/kg) in sterile phosphate‐buffered saline (20 *μ*L) was infused via the tail vein and after 30 min, the animals were killed and the thoracic cavity was opened. The lung vasculature was washed by perfusion with 100 mL of phosphate‐buffered saline (0.1 mol/L NaCl, 0.05 mol/L Phosphate, pH 7.4) via the right ventricle. The lungs were harvested, blotted, weighed, and snap frozen in liquid nitrogen. The frozen lung was pulverized in tissue homogenizer and incubated in dimethyl formamide overnight at 37°C. The concentration of EB was determined by spectrophotometry using an excitation at 620 nm and emission at 680 nm wavelength 13, and expressed as Evans blue per gram of lung tissue. To test the effect of ROCK1 deficiency, in separate experiments, control and ROCK^−/−^ FVB mice were given intraperitoneal injection of dasatinib 10 mg/kg daily for 5 days. On the sixth day, Evans blue 2 mg/kg in PBS (20 *μ*L) was infused via the tail vein and after 30 min, the animals were killed and extravascular Evans blue was determined as above.

## Results

### Increased permeability of dasatinib‐treated microvascular endothelial cells

**Figure 1:**
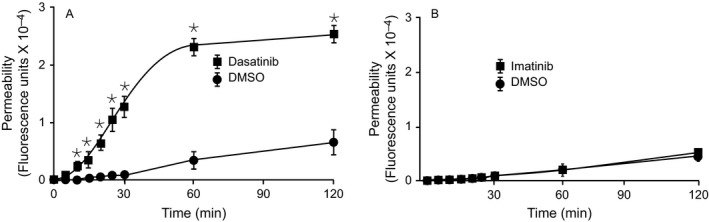
**Effect of dasatinib on endothelial permeability**. Human microvascular endothelial cells‐1 were seeded on Transwell insert plates (membrane pore size: 3 *μ*m) and after confluence fluorescein isothiocyante (FITC)‐dextran (1.0 mg/mL) was loaded to the apical chamber and the cells were treated with 100 ng/mL dasatinib (A) or Imatinib (B). At various times, the lower chamber was sampled for the presence of FITC‐dextran. Results are mean and SD of quadruplicate wells of typical experiments. *P value of < 0.05 or less for dasatinib versus dimethyl sulfoxide (vehicle).

Using monolayers of HMEC‐1 in a Transwell apparatus, we tested the effect of therapeutic concentration of dasatinib (100 ng/mL) on the barrier function by evaluating the permeability to FITC‐dextran. As seen in Figure 1A, transendothelial transport of FITC‐dextran across the endothelial cell monolayer was increased within 5 min following the addition of dasatinib compared to the untreated endothelial cells. The maximum effect was seen within an hour. Under similar conditions, imatinib had no effect on permeability (Fig. 1B) even at a concentration of 400 ng/mL (data not shown).

### Activation of RhoA/ROCK pathway by dasatinib

**Figure 2:**
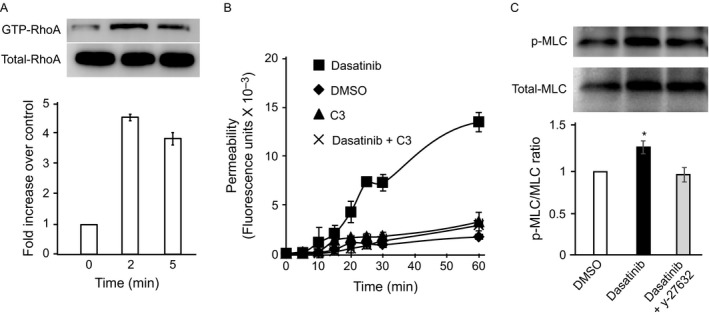
**RhoA activation in dasatinib‐treated cells.** (A) Rhotekin‐linked agarose beads were used to pull down Rho‐GTP from lysates of human microvascular endothelial cells‐1 (HMEC‐1) at indicated times after dasatinib incubation. The samples were then immunoblotted with anti‐RhoA Ab. (B) Effect of RhoA inhibition on endothelial permeability. HMEC‐1 were seeded onto Transwell insert and incubated with vehicle [dimethyl sulfoxide (DMSO)], dasatinib or the combination of dasatinib and the RhoA inhibitor I, C3. At various times, the lower chamber was sampled and analyzed for the presence of fluorescein isothiocyante‐dextran. Results are mean and SD of quadruplicate wells of typical experiments. (C) HMEC‐1 were incubated with dasatinib (100 ng/mL) and the cells were solubilized. Lysates were subjected to SDS‐PAGE, electrophoretically transferred to PVDF membranes and immunoblotted with antibodies to total and Phospho‐myosin light chain. The ratios of relative band intensities (means and SD) of three independent experiments are shown. * denotes a *P* value of less than 0.05 compared to DMSO.

The small GTPase RhoA primarily regulates the cytoskeletal organization that controls the endothelial permeability. We measured the effect of dasatinib on RhoA activation. Dasatinib induced a rapid activation of RhoA in monolayers of HMEC‐1 (Fig. 2A). Consistent with the ability of dasatinib to induce RhoA activation, RhoA inhibitor I (C3 transferase) blocked dasatinib‐induced permeability (Fig. 2B). Two closely related kinases, Rho‐associated coiled‐coil serine/threonine kinase‐1 (ROCK1) and 2 (ROCK2) have been identified as key downstream effectors of RhoA. ROCK kinases enhance MLC activity by directly phosphorylating MLC on threonine 18/19 and/or inhibiting the myosin phosphatase. Phosphorylation of MLC also promotes myosin ATPase activity and facilitates actomyosin contraction and loss of barrier function. Therefore, we evaluated the effect of dasatinib on MLC phosphorylation. Compared to the DMSO treatment, dasatinib treatment resulted in a moderate but significant increase in MLC phosphorylation, which was attenuated by the ROCK inhibitor y27632 (Fig. 2C). Under similar conditions, imatinib had no effect on RhoA activation (data not shown).

### Dasatinib alters the spreading and cytoskeletal organization of microvascular endothelial cells

**Figure 3:**
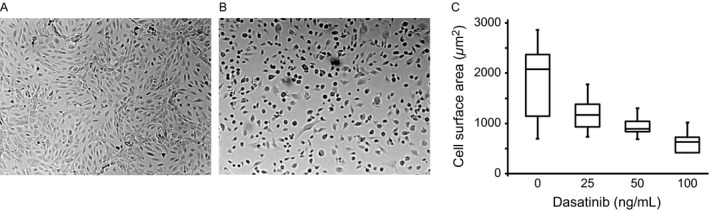
**Effect of dasatinib on endothelial cell morphology.** Human microvascular endothelial cells‐1 were grown on fibronectin‐coated coverslips. Following confluence (24–48 h), the cells were incubated with dimethyl sulfoxide (A) or dasatinib (B) for 60 min, and examined under microscope (100×). In (C), cell suspensions were incubated with various concentrations of dasatinib (60 min), allowed to adhere to fibronectin‐coated plates for 30 min at 37°C. The cells were fixed, stained with eosin, and cell surface area was quantified by ImageJ software.

**Figure 4:**
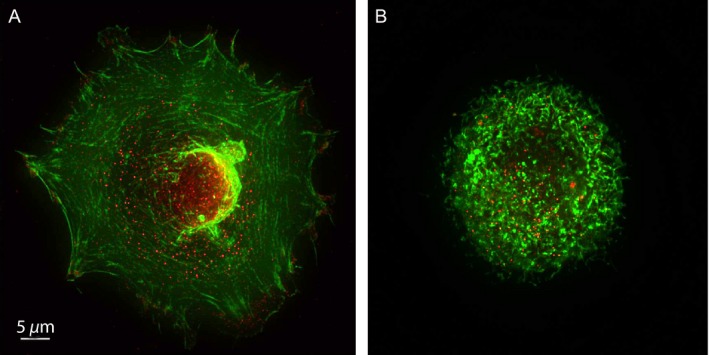
**Effect of dasatinib on endothelial cell actin fiber organization.** Subconfluent human microvascular endothelial cells‐1 were treated with dimethyl sulfoxide (A) or dasatinib (B), fixed, permeabilized and stained with phalloıdin (green) for F‐actin or FAK (red) for adhesion plaques. Images were taken in a confocal microscope (1000X).

The cultured HMEC‐1 have the morphology of regular, polygonal cells densely packed as a continuous monolayer when plated on fibronectin. Following incubation with dasatinib, the cells became granular, with retraction from the fibronectin‐coated substrate. (Fig. 3A and B). The tight cellular organization of the endothelial monolayer and the stretched morphologies of individual cells were impaired and the cells were shrunken and more rounded. Quantification of endothelial cell spreading on fibronectin substrate was performed by measuring the surface area using imageJ software 12 following the exposure to various concentrations of dasatinib. Dasatinib inhibited the spreading of these cells in a concentration‐dependent manner (Fig. 3C). Cell spreading is maintained by the counterbalance between the contractile forces generated by the actin cytoskeleton with the resistance generated by the interactions between the cell surface integrins and their substrates in the extracellular matrix. Therefore, we examined the cytoskeletal organization of endothelial cells after exposure to dasatinib by labeling F‐actin with phalloidin in a confocal microscope. As shown in the fluorescent images (Fig. 4A and B), nonchallenged endothelial cells had well‐formed actin fibers with focal adhesion plaques. Following dasatinib treatment, the actin fibers became disorganized and irregular. The stretched morphology of the cells was lost despite the presence of focal adhesion plaques, and the actin fibers were not congruous with focal adhesion plaques.

### Dasatinib inhibits tyrosine phosphorylation of p130cas, paxillin, and vinculin

**Figure 5:**
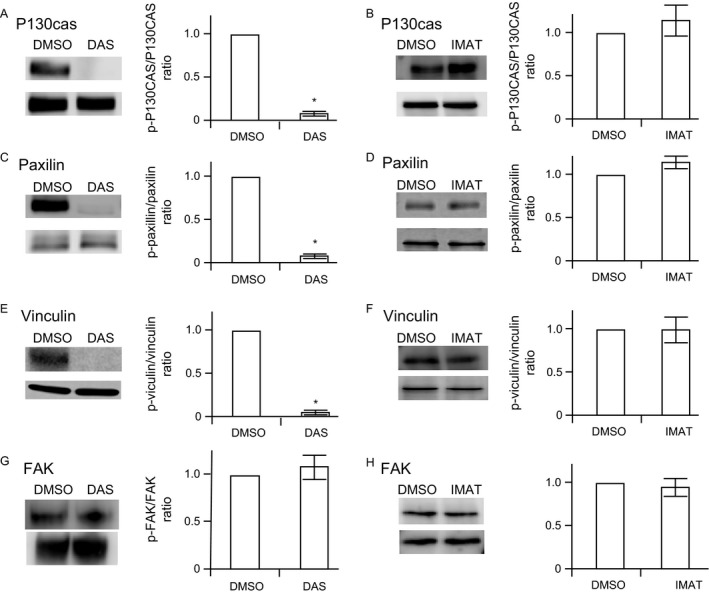
**Effect of dasatinib on tyrosine phosphorylations of p130cas, paxillin, vinculin and,** **FAK** **.** Human microvascular endothelial cells‐1 were incubated with 100 ng/mL of dasatinib (DAS) (panels A, C, E, G), or imatinib (IMAT) (panels B, D, F, H) and the cells were solubilized. Lysates were subjected to SDS‐PAGE, electrophoretically transferred to PVDF membranes and immunoblotted with phosphospecific antibodies to p130cas, paxillin, or vinculin. The blots were stripped of antibodies and reprobed with corresponding antibodies to total protein. The relative intensity of phosphoblot to the corresponding total protein in dimethyl sulfoxide (DMSO)‐treated cells was considered as 1 for comparison. The phosphorylation status of FAK was assessed by immunoprecipitation from cell lysate with FAK antibody and probed with phosphotyrosine and FAK antibodies. A representative blot and ratios of relative band intensities (means and SD) of three independent experiments are shown. * denotes a *P* value of less than 0.05 compared to DMSO.

**Figure 6:**
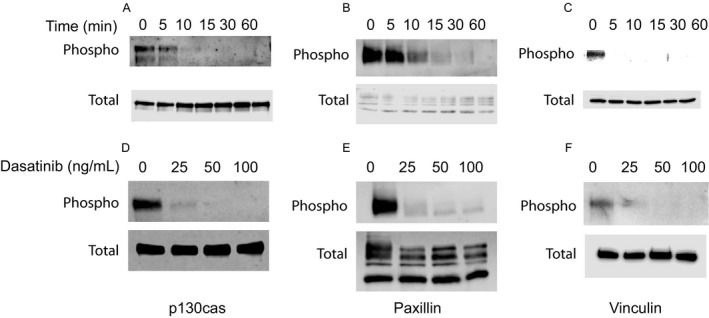
**Time and concentration‐dependent effect of dasatinib on tyrosine phosphorylations of p130cas, paxillin, and vinculin**. (A–C) Human microvascular endothelial cells (HMEC‐1) were incubated with dasatinib 100 ng/mL for the indicated times (0–60 min) and the cells were solubilized, and subjected to SDS‐PAGE, electrophoretically transferred to PVDF membranes and immunoblotted with antibodies to phosphospecific and total p130cas, paxillin, or vinculin. (D–F) HMEC‐1 were incubated with various concentrations of dasatinib and immunoblotted with phosphospecific or total p130cas, paxillin, or vinculin.

The changes in the cytoskeletal organization suggested alterations in the functional status of cytoskeletal proteins involved in cell spreading. Since dasatinib is tyrosine kinase inhibitor and tyrosine phosphorylation plays a major role in the maintenance of cytoskeleton, we examined phosphorylation status of several cytoskeletal proteins. Tyrosine phosphorylation of p130cas (Y165), paxillin (Y118), and vinculin (Y1065) were markedly decreased by dasatinib (Fig. 5A, C and E). Under similar conditions, imatinib had no effect on tyrosine phosphorylation of these proteins at 100 ng/mL (Fig. 5B, D, and F). Under the similar conditions, the phosphorylation of FAK was not significantly affected by dasatinib or imatinib (Fig. 5G and H). The effect of dasatinib is time and concentration‐dependent and it is seen within 5–10 min of incubation (Fig. 6).

### Pharmacological inhibition and genetic ablation of ROCK1 attenuate dasatinib‐induced microvascular permeability

**Figure 7:**
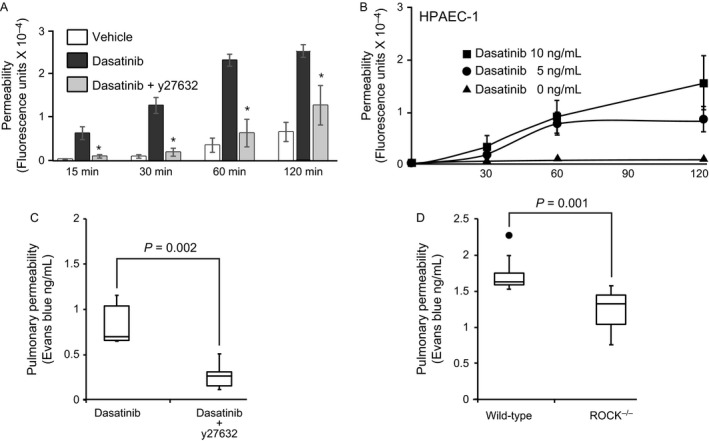
**Effect of ROCK inhibition on endothelial permeability**. (A) Human microvascular endothelial cells‐1 were seeded onto Transwell insert and incubated with vehicle (dimethyl sulfoxide), dasatinib, or the combination of dasatinib and the ROCK inhibitor, y27632. At various times, the lower chamber was sampled and analyzed for the presence of fluorescein isothiocyante(FITC)‐dextran. Results are mean and SD of four experiments. * indicates a *P* value of < 0.05 versus dasatinib. (B) Human pulmonary artery endothelial cells were seeded on Transwell insert plates as in (A) and after confluence FITC‐dextran (1.0 mg/mL) was loaded to the apical chamber and the cells were treated with 0, 5, or 10 ng/mL dasatinib. At various times, the lower chamber was sampled for the presence of FITC‐dextran. (C) Wild‐type mice were treated with saline or y27632 (*N* = 5 in each group) and after 60 min, dasatinib (10 mg/kg) was given intraperitoneally and 4 h later Evans blue (2 mg/kg) was injected intravenously. The animals were killed after 30 min and the extravascular Evans blue in pulmonary parenchyma was quantified spectrophotometrically. (D) Effect of dasatinib in ROCK1‐deficient mice. In separate set of experiments, dasatinib‐treated wild‐type and ROCK1‐deficient mice (*N* = 12 in each group) were given Evans blue intravenously and killed after 30 min. The extravascular Evans blue in pulmonary parenchyma was quantified as in (C).

Since dasatinib treatment led to the activation of RhoA and MLC (Fig. 2), we evaluated whether inhibition of Rho‐ROCK‐MLC signaling axis would improve the barrier function of dasatinib‐treated endothelial cell monolayers. Therefore, we assessed endothelial permeability in the presence of ROCK1 inhibitor. Dasatinib‐induced increase in endothelial permeability was blocked by y27632 (Fig. 7A). To extend the key observation of dasatinib‐induced changes in HMEC‐1, we investigated the barrier function in an independent physiologically relevant human pulmonary artery endothelial cells (HPAEC). Dasatinib at concentration as low as 5–10 ng/mL promoted permeability in HPAEC (Fig. 7B).

To explore further, the in vivo significance of ROCK inhibition in ameliorating dasatinib‐induced endothelial cell permeability, we administered dasatinib (10 mg/kg) intraperitoneally in mice and after 4 h, assessed pulmonary microvascular permeability by measuring the extravasation of intravenously administered Evans blue 30 min before killing 13. Dasatinib‐induced extravasation of Evans blue into the pulmonary parenchyma was reduced significantly by the prior administration of y27632 (Fig. 7C) showing that the attenuating effect of ROCK inhibitor on dasatinib‐induced permeability was also observed in vivo. Furthermore, in ROCK1‐deficient mice, dasatinib‐induced pulmonary microvascular permeability was attenuated compared to their wild‐type controls (Fig. 7D). These observations, taken together with studies with cultured endothelial cells, indicate that dasatinib promoted endothelial permeability via the Rho‐ROCK‐MLC pathway and ROCK inhibitor attenuated the endothelial dysfunction.

## Discussion

Our results show that therapeutic concentrations of dasatinib impair the barrier function of monolayers of HMEC‐1 by activating the RhoA/ROCK pathway. RhoA cycles between inactive GDP‐bound form and an active GTP‐bound form. The activation of RhoA results in increased actomyosin contractility due to myosin light chain phosphorylation. Excessive activation of RhoA causes an increase in the contractile force that pulls the endothelial cells and disrupts the barrier function. RhoA mediates its effect by binding to downstream effectors and two closely related kinases, Rho‐associated coiled‐coil serine/threonine kinase‐1 (ROCK1) and ‐2 (ROCK2). These kinases have been identified as key downstream effectors of RhoA. ROCK has previously been shown to induce VEGF 14, thrombin 15, TGF‐*β* 16, and thermal injury 17 mediated increases in vascular permeability by promoting actin filament retraction by phosphorylating downstream target proteins including myosin light chains 18. ROCK can be activated either by RhoA GTPase or by proteolytic cleavage of inhibitory carboxyl‐terminal domain by caspase‐3 during apoptosis 19, 20. However, there was no proteolytic cleavage of ROCK1 in dasatinib‐treated cells (data not shown), consistent with RhoA activation of ROCK1 as the primary mechanism.

Upon engagement with its ligands, integrins cluster and recruit various signaling and adaptor proteins to form focal adhesions. Strategically localized, focal adhesion plaques are positioned to modulate the signaling pathways that maintain actin fibers 21, 22. Focal adhesions contain over 150 different proteins, which suggest a considerable functional diversity. Integrins, an essential component of focal adhesions, form a functional and mechanical link between intracellular actin fibers and the extracellular matrix. The cytoplasmic domains of integrins interact with components of cytoskeleton in focal adhesion plaques, and transmit inhibitory signals to RhoA 23, 24, 25. Swiss 3T3 fibroblasts show inhibition of RhoA activation when plated on fibronectin 26. Adherent cells had lower RhoA activation than cells in suspension consistent with an adhesion‐dependent negative feedback. Furthermore, shear stress induces conformational activation of integrins, which suppresses RhoA activity in endothelial cells 27. Tyrosine kinases play an essential role in transmitting inhibitory signals from integrins to RhoA 28, 29. Dasatinib, a tyrosine kinase inhibitor, markedly reduces tyrosine phosphorylation of p130cas, paxillin, and vinculin, which are localized to the actin cytoskeleton in the focal adhesions. P130cas, a prominent Src substrate, is a major component of focal adhesion and it acts as a mechanosensor of force 24. Mechanical stretch extends the substrate domain of p130cas, unmasking tyrosine phosphorylation motifs. Tyrosine phosphorylation of p130cas by src kinases provides binding sites for FAK 30, 31. P130cas‐deficient fibroblasts exhibited thin, short, and irregularly assembled actin filaments at the cell periphery with normal focal adhesions and diminished spreading on fibronectin substrate, very similar to dasatinib‐treated endothelial cells 32. Transfection of p130cas‐deficient fibroblasts with wild‐type p130cas rescues the actin fibers assembly/disassembly, while transfection with variants lacking the phosphorylation sites had no effect on abnormal actin organization 33. These studies show a central role for p130cas phosphorylation in maintaining the actin network. Paxillin, a focal adhesion adaptor protein of ~70 kDa, is also an important scaffolding protein at focal adhesions that binds to the cytoplasmic tail of integrins and numerous signaling molecules including adaptor molecules p130cas, FAK, and SRC 34, 35. Vinculin, a 117‐kDa protein provides binding sites for talin, F‐actin, and paxillin. Both paxillin‐ 36, 37 and vinculin 38‐deficient cells show reduced spreading on fibronectin substrate similar to dasatinib‐treated endothelial cells. By inhibiting tyrosine phosphorylation of at least three cytoskeletal components (p130cas, paxillin, and vinculin) of the focal adhesion, dasatinib disrupts flow of inhibitory signals from integrins to RhoA, leading to the activation of RhoA/ROCK pathway. Interestingly, the phosphorylation of FAK is not significantly affected by dasatinib suggesting that the ability of dasatinib to modulate endothelial cytoskeleton is independent of FAK phosphorylation. ROCK inhibition ameliorates dasatinib‐induced microvascular permeability in vitro and in vivo. There are limitations to this study in that the findings are confined to mice. Human endothelial cells in vivo may not respond similarly. Furthermore, in dasatinib therapy, permeability changes are primarily seen in pulmonary vasculature, while alterations in phosphorylation will be expected to occur in all blood vessels. Imatinib is more specific inhibitor of BCL‐ABR kinase and does not inhibit other SFKs 39 and phosphorylation of p130cas, paxillin, or vinculin was not altered by imatinib.

In summary, our results show that dasatinib inhibits tyrosine phosphorylation of cytoskeletal proteins, p130cas, paxillin, and vinculin (and possibly many other tyrosine kinase substrates) leading to the activation of RhoA/ROCK pathway. ROCK inhibitors, such as fasudil, are in clinical trial for pulmonary hypertension and our studies suggest a potential beneficial effect of ROCK1 inhibitor for patients on dasatinib who develop peripheral edema, pleural pulmonary edema, and pulmonary hypertension.

## Conflict of Interest

The authors declare no conflict of interest.

## References

[1] Druker, B. J. 2008 Translation of the Philadelphia chromosome into therapy for CML. Blood 112:4808–4817. 19064740 10.1182/blood-2008-07-077958

[2] Ernst, T. , P. La Rosee , M. C. Muller , and A. Hochhaus . 2011 BCR‐ABL mutations in chronic myeloid leukemia. Hematol. Oncol. Clin. North Am. 25:997–1008, v–vi. 22054731 10.1016/j.hoc.2011.09.005

[3] Lindauer, M. , and A. Hochhaus . 2014 Dasatinib. Recent Results Cancer Res. 201:27–65. 24756784 10.1007/978-3-642-54490-3_2

[4] Lombardo, L. J. , F. Y. Lee , P. Chen , D. Norris , J. C. Barrish , K. Behnia , et al. 2004 Discovery of N‐(2‐chloro‐6‐methyl‐ phenyl)‐2‐(6‐(4‐(2‐hydroxyethyl)‐ piperazin‐1‐yl)‐2‐methylpyrimidin‐4‐ ylamino)thiazole‐5‐carboxamide (BMS‐354825), a dual Src/Abl kinase inhibitor with potent antitumor activity in preclinical assays. J. Med. Chem. 47:6658–6661. 15615512 10.1021/jm049486a

[5] Krauth, M. T. , S. Herndlhofer , M. T. Schmook , G. Mitterbauer‐Hohendanner , E. Schlogl , and P. Valent . 2011 Extensive pleural and pericardial effusion in chronic myeloid leukemia during treatment with dasatinib at 100 mg or 50 mg daily. Haematologica 96:163–166. 20934998 10.3324/haematol.2010.030494 PMC3012781

[6] Quintas‐Cardama, A. , H. Kantarjian , S. O'Brien , G. Borthakur , J. Bruzzi , R. Munden , et al. 2007 Pleural effusion in patients with chronic myelogenous leukemia treated with dasatinib after imatinib failure. J. Clin. Oncol. 25:3908–3914. 17761974 10.1200/JCO.2007.12.0329

[7] Alexander, J. S. 2000 Rho, tyrosine kinase, Ca(2+), and junctions in endothelial hyperpermeability. Circ. Res. 87:268–271. 10948058 10.1161/01.res.87.4.268

[8] Rizzo, A. N. , J. Aman , G. P. van Nieuw Amerongen , and S. M. Dudek . 2015 Targeting Abl kinases to regulate vascular leak during sepsis and acute respiratory distress syndrome. Arterioscler. Thromb. Vasc. Biol. 35:1071–1079. 25814671 10.1161/ATVBAHA.115.305085 PMC4655821

[9] Birch, M. , P. E. Morgan , S. Handley , A. Ho , R. Ireland , and R. J. Flanagan . 2013 Simple methodology for the therapeutic drug monitoring of the tyrosine kinase inhibitors dasatinib and imatinib. Biomed. Chromatogr. 27:335–342. 22886846 10.1002/bmc.2796

[10] Ades, E. W. , F. J. Candal , R. A. Swerlick , V. G. George , S. Summers , D. C. Bosse , et al. 1992 HMEC‐1: establishment of an immortalized human microvascular endothelial cell line. J. Invest. Dermatol. 99:683–690. 1361507 10.1111/1523-1747.ep12613748

[11] Zhang, Y. M. , J. Bo , G. E. Taffet , J. Chang , J. Shi , A. K. Reddy , et al. 2006 Targeted deletion of ROCK1 protects the heart against pressure overload by inhibiting reactive fibrosis. FASEB J. 20:916–925. 16675849 10.1096/fj.05-5129com

[12] Hartig, S. M. 2013 Basic image analysis and manipulation in ImageJ. Curr. Protoc. Mol. Biol. Chapter 14(Unit14):15. 10.1002/0471142727.mb1415s102 23547012

[13] Saria, A. , and J. M. Lundberg . 1983 Evans blue fluorescence: quantitative and morphological evaluation of vascular permeability in animal tissues. J. Neurosci. Methods 8:41–49. 6876872 10.1016/0165-0270(83)90050-x

[14] Sun, H. , J. W. Breslin , J. Zhu , S. Y. Yuan , and M. H. Wu . 2006 Rho and ROCK signaling in VEGF‐induced microvascular endothelial hyperpermeability. Microcirculation 13:237–247. 16627366 10.1080/10739680600556944

[15] Carbajal, J. M. , M. L. Gratrix , C. H. Yu , and R. C. Jr Schaeffer . 2000 ROCK mediates thrombin's endothelial barrier dysfunction. Am. J. Physiol. Cell Physiol. 279:C195–C204. 10898731 10.1152/ajpcell.2000.279.1.C195

[16] Clements, R. T. , F. L. Minnear , H. A. Singer , R. S. Keller , and P. A. Vincent . 2005 RhoA and Rho‐kinase dependent and independent signals mediate TGF‐beta‐induced pulmonary endothelial cytoskeletal reorganization and permeability. Am. J. Physiol. Lung Cell. Mol. Physiol. 288:L294–L306. 15475381 10.1152/ajplung.00213.2004

[17] Tinsley, J. H. , N. R. Teasdale , and S. Y. Yuan . 2004 Myosin light chain phosphorylation and pulmonary endothelial cell hyperpermeability in burns. Am. J. Physiol. Lung Cell. Mol. Physiol. 286:L841–L847. 14672924 10.1152/ajplung.00341.2003

[18] Dudek, S. M. , and J. G. Garcia . 2001 Cytoskeletal regulation of pulmonary vascular permeability. J. Appl. Physiol. (1985) 91:1487–1500. 11568129 10.1152/jappl.2001.91.4.1487

[19] Coleman, M. L. , E. A. Sahai , M. Yeo , M. Bosch , A. Dewar , and M. F. Olson . 2001 Membrane blebbing during apoptosis results from caspase‐mediated activation of ROCK I. Nat. Cell Biol. 3:339–345. 11283606 10.1038/35070009

[20] Sebbagh, M. , C. Renvoize , J. Hamelin , N. Riche , J. Bertoglio , and J. Breard . 2001 Caspase‐3‐mediated cleavage of ROCK I induces MLC phosphorylation and apoptotic membrane blebbing. Nat. Cell Biol. 3:346–352. 11283607 10.1038/35070019

[21] Parsons, J. T. , A. R. Horwitz , and M. A. Schwartz . 2010 Cell adhesion: integrating cytoskeletal dynamics and cellular tension. Nat. Rev. Mol. Cell Biol. 11:633–643. 20729930 10.1038/nrm2957 PMC2992881

[22] Oakes, P. W. , and M. L. Gardel . 2014 Stressing the limits of focal adhesion mechanosensitivity. Curr. Opin. Cell Biol. 30:68–73. 24998185 10.1016/j.ceb.2014.06.003 PMC4459577

[23] Harburger, D. S. , and D. A. Calderwood . 2009 Integrin signalling at a glance. J. Cell Sci. 122:159–163. 19118207 10.1242/jcs.018093 PMC2714413

[24] Janostiak, R. , A. C. Pataki , J. Brabek , and D. Rosel . 2014 Mechanosensors in integrin signaling: the emerging role of p130Cas. Eur. J. Cell Biol. 93:445–454. 25062607 10.1016/j.ejcb.2014.07.002

[25] Marjoram, R. J. , E. C. Lessey , and K. Burridge . 2014 Regulation of RhoA activity by adhesion molecules and mechanotransduction. Curr. Mol. Med. 14:199–208. 24467208 10.2174/1566524014666140128104541 PMC3929014

[26] Wang, R. , R. A. Clark , D. F. Mosher , and X. D. Ren . 2005 Fibronectin's central cell‐binding domain supports focal adhesion formation and Rho signal transduction. J. Biol. Chem. 280:28803–28810. 15964831 10.1074/jbc.M501421200

[27] Tzima, E. , M. A. del Pozo , S. J. Shattil , S. Chien , and M. A. Schwartz . 2001 Activation of integrins in endothelial cells by fluid shear stress mediates Rho‐dependent cytoskeletal alignment. EMBO J. 20:4639–4647. 11532928 10.1093/emboj/20.17.4639 PMC125600

[28] Arthur, W. T. , L. A. Petch , and K. Burridge . 2000 Integrin engagement suppresses RhoA activity via a c‐Src‐dependent mechanism. Curr. Biol. 10:719–722. 10873807 10.1016/s0960-9822(00)00537-6

[29] Sreenivasappa, H. , S. P. Chaki , S. M. Lim , J. P. Trzeciakowski , M. W. Davidson , G. M. Rivera , et al. 2014 Selective regulation of cytoskeletal tension and cell‐matrix adhesion by RhoA and Src. Integr. Biol. (Camb) 6:743–754. 24984203 10.1039/c4ib00019f

[30] Kira, M. , S. Sano , S. Takagi , K. Yoshikawa , J. Takeda , and S. Itami . 2002 STAT3 deficiency in keratinocytes leads to compromised cell migration through hyperphosphorylation of p130(cas). J. Biol. Chem. 277:12931–12936. 11812786 10.1074/jbc.M110795200

[31] Ruest, P. J. , N. Y. Shin , T. R. Polte , X. Zhang , and S. K. Hanks . 2001 Mechanisms of CAS substrate domain tyrosine phosphorylation by FAK and Src. Mol. Cell. Biol. 21:7641–7652. 11604500 10.1128/MCB.21.22.7641-7652.2001 PMC99935

[32] Honda, H. , H. Oda , T. Nakamoto , Z. Honda , R. Sakai , T. Suzuki , et al. 1998 Cardiovascular anomaly, impaired actin bundling and resistance to Src‐induced transformation in mice lacking p130Cas. Nat. Genet. 19:361–365. 9697697 10.1038/1246

[33] Meenderink, L. M. , L. M. Ryzhova , D. M. Donato , D. F. Gochberg , I. Kaverina , and S. K. Hanks . 2010 P130Cas Src‐binding and substrate domains have distinct roles in sustaining focal adhesion disassembly and promoting cell migration. PLoS ONE 5:e13412. 20976150 10.1371/journal.pone.0013412 PMC2956669

[34] Zaidel‐Bar, R. , R. Milo , Z. Kam , and B. Geiger . 2007 A paxillin tyrosine phosphorylation switch regulates the assembly and form of cell‐matrix adhesions. J. Cell Sci. 120:137–148. 17164291 10.1242/jcs.03314

[35] Brown, M. C. , and C. E. Turner . 2004 Paxillin: adapting to change. Physiol. Rev. 84:1315–1339. 15383653 10.1152/physrev.00002.2004

[36] Wade, R. , J. Bohl , and S. Vande Pol . 2002 Paxillin null embryonic stem cells are impaired in cell spreading and tyrosine phosphorylation of focal adhesion kinase. Oncogene 21:96–107. 11791180 10.1038/sj.onc.1205013

[37] Hagel, M. , E. L. George , A. Kim , R. Tamimi , S. L. Opitz , C. E. Turner , et al. 2002 The adaptor protein paxillin is essential for normal development in the mouse and is a critical transducer of fibronectin signaling. Mol. Cell. Biol. 22:901–915. 11784865 10.1128/MCB.22.3.901-915.2002 PMC133539

[38] Volberg, T. , B. Geiger , Z. Kam , R. Pankov , I. Simcha , H. Sabanay , et al. 1995 Focal adhesion formation by F9 embryonal carcinoma cells after vinculin gene disruption. J. Cell Sci. 108 (Pt. 6):2253–2260. 7673345 10.1242/jcs.108.6.2253

[39] Schindler, T. , W. Bornmann , P. Pellicena , W. T. Miller , B. Clarkson , and J. Kuriyan . 2000 Structural mechanism for STI‐571 inhibition of abelson tyrosine kinase. Science 289:1938–1942. 10988075 10.1126/science.289.5486.1938

